# Induction of apoptosis in infantile hemangioma endothelial cells by propranolol

**DOI:** 10.3892/etm.2013.1159

**Published:** 2013-06-14

**Authors:** JUN-BO TU, RUI-ZHAO MA, QIANG DONG, FEI JIANG, XIAO-YI HU, QUAN-YAN LI, PARUKJAN PATTAR, HAO ZHANG

**Affiliations:** 1 Department of Oral and Maxillofacial Surgery, Stomatological Hospital, Xi’an Jiaotong University, Xi’an, Shaanxi 710004, P.R. China; 2 Department of Oral and Maxillofacial Surgery, Weihai Stomatological Hospital, Weihai, Shandong 264200, P.R. China

**Keywords:** hemangioma, apoptosis, mitochondria, mechanism, propranolol

## Abstract

Propranolol, a non-selective β-blocker, is emerging as an effective treatment for complicated hemangiomas. The aim of this study was to investigate the molecular mechanism(s) underlying the therapeutic effects of propranolol against hemangiomas, using primary infantile hemangioma endothelial cells (IHECs). IHECs were treated with various concentrations of propranolol and morphological changes and apoptosis were assessed. Changes in the expression levels of apoptosis-related genes were examined. Annexin-V staining revealed that propranolol at 40, 50 and 60 *μ*g/ml caused a concentration-dependent increase in the apoptosis of IHECs. Morphological analyses revealed that exposure to 50 *μ*g/ml propranolol resulted in typical apoptotic changes, including shrinkage, the formation of apoptotic bodies and retention of plasma membrane integrity. Gene expression analyses revealed that propranolol treatment led to a marked increase in the expression of caspase-8, cytochrome *c*, apoptosis-inducing factor, caspase-3 and poly (ADP-ribose) polymerase 1, as well as a concomitant reduction in lamin B1 expression. Our data collectively demonstrate that propranolol induces apoptosis of IHECs through activation of the intrinsic and extrinsic apoptotic pathways, which represents an important mechanism for its therapeutic effects against infantile hemangiomas.

## Introduction

Hemangioma is the most common benign tumor of infancy. The incidence in newborns is 2–3% and this increases to ∼10% by the age of 1 year (1). Typically, hemangioma lesions appear in the early postnatal period (1–2 weeks), rapidly proliferate for up to 12 months and gradually involute over 5–10 years (2,3). Infantile hemangioma has been suggested to arise from the clonal expansion of endothelial cells, based on the analysis of X-chromosome inactivation patterns (2,4). It may also be a result of somatic mutations in one or more genes that affect endothelial cell growth (5). However, the pathogenesis of hemangiomas remains largely unknown. Although the majority of hemangiomas are benign and resolve spontaneously, ∼10% may cause cosmetic and life-threatening complications and thus require treatment (6).

Several pharmacological therapies are currently available for patients with problematic hemangiomas, including steroids, interferon-α, bleomycin, vincristine and β-blockers (7,8). Induction of apoptosis represents an important mechanism for drug-induced hemangioma regression (9). Generally, there are two main apoptotic pathways: The extrinsic or death receptor pathway and the intrinsic or mitochondrial pathway (10,11). The two pathways converge on the activation of caspases, which comprise a family of cysteine proteases and play a central role in the execution of apoptosis. In the mitochondrial-initiated pathway, caspase activation is triggered by the formation of a multimeric Apaf-1/cytochrome *c* complex, which is implicated in the activation of procaspase-9. Activated caspase-9 then cleaves and activates downstream caspases, including caspase-3, -6 and -7, ultimately culminating in apoptosis.

Propranolol is a non-selective β-blocker commonly used in the treatment of cardiovascular diseases, including heart failure and hypertension. The therapeutic benefit of propranolol in hemangiomas was accidentally identified in 2008 by Léauté-Labrèze *et al* (12) who noted the regression of an infantile hemangioma following the administration of propranolol for steroid-induced hypertrophic cardiomyopathy. Since then, several other studies have also reported the effectiveness of propranolol in the treatment of problematic infantile hemangiomas (13,14). In the current study, we aimed to investigate the molecular mechanism(s) underlying the therapeutic effects of propranolol against hemangiomas, using primary infantile hemangioma endothelial cells (IHECs).

## Materials and methods

### Cell culture

Human IHECs isolated from a proliferating infantile hemangioma were obtained from the Department of Pediatric Surgery, Second Hospital of Xi’an Jiaotong University (Xi’an, China). Cells were cultured in RPMI-1640 medium supplemented with 10% fetal bovine serum, 10 *μ*g/ml streptomycin and 100 U/ml penicillin (Invitrogen Life Technologies, Carlsbad, CA, USA) at 37°C under 5% CO_2_. Confluent cells were routinely subcultured using trypsin-ethylenediaminetetraacetic acid (EDTA) solution (0.05%; Invitrogen Life Technologies) and cells at passages 5–6 were used in this study. The study was approved by the medical ethics committee of Xi’an Jiaotong University and informed consent was obtained from the patient’s family.

### Drug treatment

Cells were seeded in quadruplicate at a density of 1×10^5^ cells per well into 24-well plates. After incubation overnight at 37°C, cells were left untreated as the controls or treated with propranolol (Tianjin Lisheng Pharmaceutical Co., Ltd., Tianjin, China) at 40, 50 or 60 *μ*g/ml for 24 h. Morphological and biochemical changes of the cells were then examined using the following methods.

### Morphological changes detected by light microscopy

Cell morphology was examined every 1 h until 40 h after indicated treatments under a contrast-phase microscope (Leica, Bensheim, Germany).

### Transmission electron microscopy (TEM)

For TEM examination, cells were harvested 24 h after drug treatment. The cells were prefixed in 2.5% glutaraldehyde, postfixed in 1% osmium tetroxide and dehydrated in an ascending series of ethanol to 100%. The cell samples were then embedded and cut into ultrathin sections (50–70 nm). Sections were stained with 0.5% uranyl acetate and saturated lead citrate and examined using an electron microscope (H-600, Hitachi, Tokyo, Japan).

### Apoptosis analysis

After drug treatment for 24 h, cells were collected, washed and subjected to apoptosis analysis using an Annexin V-fluorescein isothiocyanate (FITC) kit (Trevigen, Gaithersburg, MD, USA), according to the manufacturer’s instructions. Cells were analyzed using a FACScan flow cytometer with CellQuest software (BD Biosciences, Franklin lakes, NJ, USA).

### Western blot analysis

The primary antibodies used were as follows: Anti-caspase-8 (1:1,000 dilution), anti-cleaved caspase-3 (1:1,000 dilution), anti-lamin B1 (1:1,000 dilution), anti-cytochrome *c* (1:500 dilution), anti-apoptosis-inducing factor (AIF; 1:500 dilution), anti-poly (ADP-ribose) polymerase 1 (PARP1; 1:1,000 dilution) and anti-glyceralde-hyde-3-phosphate dehydrogenase (GAPDH; 1:1,000 dilution). These antibodies were purchased from ProteinTech (Chicago, IL, USA). Samples of protein extracts were resolved by sodium dodecyl sulfate-polyacrylamide gel electrophoresis and transferred to nitrocellulose membranes (Pierce Biotechnology, Inc., Rockford, IL, USA). After blocking with 5% fat-free milk solution, the membranes were incubated overnight at 4°C with primary antibodies, followed by incubation with horseradish peroxidase-linked secondary antibodies (Pierce Biotechnology, Inc.) for 1 h at 37°C. Bound antibodies were visualized by an enhanced chemiluminescence detection kit (Toyobo Co., Ltd., Osaka, Japan). Signal intensities were quantitated using Quantity One Software (Bio-Rad, Hercules, CA, USA).

### Quantitative polymerase chain reaction (qPCR)

Total RNA was extracted with TRIzol according to the manufacturer’s instructions (Invitrogen Life Technologies). Reverse transcription was performed using the First Strand cDNA Synthesis kit (MBI Fermentas, Vilnius, Lithuania). qPCR amplification was conducted using the IQ5.0 Real-Time PCR System (Bio-Rad) and SYBR-Green PCR Master mix (Toyobo Co., Ltd.). The PCR primers used are listed in Table I. The PCR conditions were as follows: initial denaturation at 95°C for 1 min, followed by 40 cycles of denaturation (95°C for 15 sec), annealing (60°C for 15 sec) and extension (72°C for 45 sec) and then a last extension at 72°C for 10 min. All assays were performed in triplicate and the threshold cycle (Ct) was calculated. The relative mRNA expression level normalized by that of β-actin (used as an internal control) was then determined using the 2^−ΔΔCt^ method (15).

### Statistical analysis

Significant differences between groups were calculated using the Student’s t-test. One-way analysis of variance followed by the Tukey’s post hoc test was used to examine differences among multiple groups. P<0.05 was considered to indicate a statistically significant difference.

## Results

### Effects of propranolol on the apoptosis of IHECs

**Figure 1.**
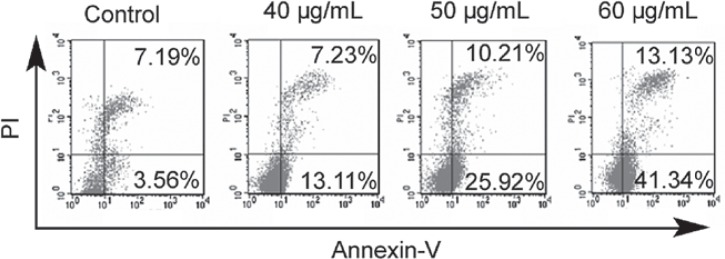
Effects of propranolol on the apoptosis of IHECs. IHECs were left untreated or exposed to 40, 50 or 60 *μ*g/ml propranolol for 24 h and then stained with Annexin-V and PI. Apoptosis was analyzed with flow cytometry. Cell populations in the upper (late apoptosis) and lower (early apoptosis) right quadrants were designated as apoptotic cells. Representative dot plots of three separate experiments with similar results are shown. IHECs, infantile hemangioma endothelial cells; PI, propidium iodide.

As shown in Fig. 1, treatment with propranolol for 24 h resulted in a significant increase in the apoptosis of IHECs, which occurred in a concentration-dependent manner. Propranolol at 60 *μ*g/ ml caused ∼3-fold more apoptotic cells than propranolol at 40 *μ*g/ml (54 vs. 20%).

### Effects of propranolol on the morphology of IHECs

**Figure 2.**
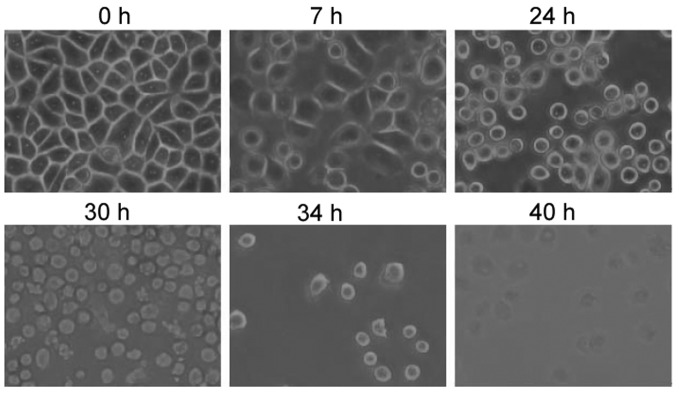
Time course of the morphological changes of IHECs treated with 50 *μ*g/ml propranolol observed using phase-contrast microscopy (Leica DM IL LED). Original magnification, ×100. IHECs, infantile hemangioma endothelial cells.

**Figure 3.**
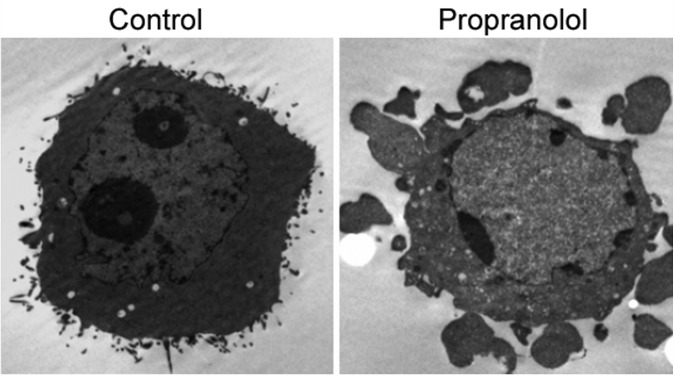
Ultrastructural characteristics of untreated IHECs and those treated with 50 *μ*g/ml propranolol for 24 h observed by transmission electron microscopy (JEM-100CXII). Magnification, ×6,000. IHECs, infantile hemangioma endothelial cells.

Morphological examination using phase-contrast microscopy revealed that the IHECs became round and a few were detached from the culture plates at 24 h after exposure to propranolol at a concentration of 50 mg/ml (Fig. 2). At 30 h after drug treatment, the IHECs presented a shrunken cytoplasm, with an intact plasma membrane, indicative of the typical morphology of apoptosis. Apoptosis was observed in the majority of IHECs exposed to propranolol for 40 h. Consistent with the results of light microscopy, TEM examination confirmed the induction of apoptosis in the IHECs treated with 50 *μ*g/ml propranolol for 24 h (Fig. 3). Control cells presented a normal morphology (Fig. 3A). By contrast, the propranolol-treated cells exhibited typical characteristics of apoptosis, including an intact cell membrane, chromatin condensation, fragmentation of nuclei and the formation of apoptotic bodies (Fig. 3B).

### Protein and mRNA levels of apoptosis-related genes

**Figure 4.**
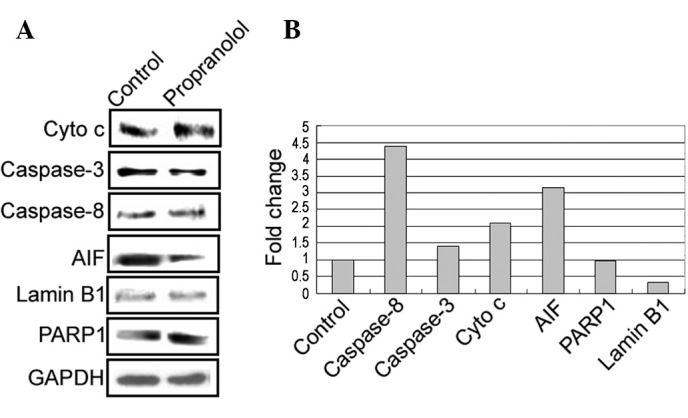
Examination of the expression levels of caspase-8, caspase-3, AIF, cytochrome *c* (Cyto *c*), PARP1 and lamin B1 in IHECs treated with or without propranolol. (A) Representative western blots of each group are shown. GAPDH was used as the loading control. (B) Analysis of the mRNA levels of indicated genes by quantitative PCR. Data are expressed as fold change relative to the control (set to 1). AIF, apoptosis-inducing factor; PARP1, poly (ADP ribose) polymerase 1; IHECs, infantile hemangioma endothelial cells; GAPDH, glyceraldehyde 3-phosphate dehydrogenase.

Western blot analysis revealed that the propranolol-treated cells had a marked increase in protein levels of caspase-8, cytochrome *c*, apoptosis-inducing factor, caspase-3 and poly (ADP-ribose) polymerase 1, as well as a concomitant reduction in lamin B1, (Fig. 4A). The qPCR assay further confirmed similar changes at the mRNA level upon exposure to propranolol (Fig. 4B).

## Discussion

Propranolol is gaining increasing attention in the treatment of infantile hemangiomas. Bertrand *et al* (13) reviewed a series of 35 consecutive patients with infantile hemangioma and reported that oral propranolol was effective in controlling the proliferative phase of problematic infantile hemangioma, without causing serious adverse effects. A meta-analysis study conducted by Izadpanah *et al* (16) revealed that propranolol achieves a greater response rate in the treatment of infantile hemangiomas in comparison with corticosteroids (99 vs. <90%). Propranolol has been reported to have the ability to block cell proliferation and migration of IHECs (17). Our results further elucidated the biological role of propranolol in IHECs and demonstrated that exposure to propranolol caused significant apoptosis in IHECs. Moreover, this pro-apoptotic effect was shown to be concentration-dependent. Apoptosis is regarded as an active suicidal response, which is characterized by nuclear condensation and fragmentation, cellular shrinkage without loss of plasma membrane integrity and the formation of apoptotic bodies. Forty hours after treatment with a moderate dose (50 *μ*g/ml) of propranolol, almost all the cells were observed to go apoptosis. These data provide an explanation for the excellent performance of propranolol in the treatment of infantile hemangiomas. Propranolol-induced apoptosis of hemangioma endothelial cells has been documented in previous studies (18,19).

It is well accepted that apoptosis is induced by two distinct pathways, extrinsic and intrinsic (20). The extrinsic pathway involves the ligation of death receptors, the activation of caspase-8 and the cleavage and activation of effector caspases, particularly caspase-3. The intrinsic signaling pathways involve a diverse array of non-receptor-mediated stimuli, which initiate different intracellular signal cascades that converge at the level of the mitochondria, leading to mitochondrial membrane permeabilization and the release of pro-apoptotic factors, including cytochrome *c* and AIF, into the cytoplasm. Propranolol has been shown to exert growth-suppressive effects on pancreatic cancer cells through the induction of caspase-9-and caspase-3-mediated apoptosis (17). Similarly, Ji *et al* (19) demonstrated that treatment with propranolol induces apoptosis in hemangioma-derived endothelial cells, which involves the activation of caspase-9 and caspase-3 of the intrinsic pathway. In agreement with these observations, our data revealed that treatment with propranolol initiated the intrinsic apoptotic pathway in IHECs, as shown by the increased release of cytochrome *c* and AIF into the cytoplasm and the enhanced cleavage of caspase-3 and PARP1. Moreover, propranolol treatment also increased the level of caspase-8, suggesting an involvement of the extrinsic apoptotic pathway. Lamin B1 is a member of the nuclear lamin family of proteins and is thought to be involved in nuclear stability and chromatin structure. Lamin B1 degradation is recognized as an early feature of apoptosis (21). Our data indicated a marked reduction of the levels of lamin B1 following propranolol treatment, suggesting that the downregulation of this gene is involved in propranolol-induced apoptosis.

However, there are a number of limitations of the present study that should be noted. Firstly, the detailed signaling pathways involved in the propranolol-induced apoptosis of IHECs continue to require definition. Additionally, it remains unclear whether the findings of this study may be translated into an *in vivo* setting.

The induction of apoptosis has been regarded as a preferred and superior strategy for clearing tumor cells. The retention of plasma membrane integrity during apoptosis prevents the onset of an inflammatory response that favors tumor progression (22). Our data collectively demonstrate that propranolol is capable of inducing apoptosis in IHECs through activation of the intrinsic and extrinsic apoptotic pathways, which represents an important mechanism for its therapeutic effects against infantile hemangiomas.

## Figures and Tables

**Table I.** Primers used for quantitative PCR assays.

Gene	Primer sequence
Cytochrome *c*	Sense: GGTGATGTTGAGAAAGGCAAGAAG
	Antisense: GGCGGCTGTGTAAGAGTATCC
Caspase-3	Sense: CTGGACTGTGGCATTGAGAC
	Antisense: ACAAAGCGACTGGATGAACC
Caspase-8	Sense: ATTAGGGACAGGAATGGAACACAC
	Antisense: GGAGAGGATACAGCAGATGAAGC
AIF	Sense: CCAGGCAACTTGTTCCAG
	Antisense: TTCATAGTCTTGTAGGCATAGG
Lamin B1	Sense: AATCGTTGTCAGAGCCTTAC
	Antisense: CCTTATACAGCCTCACTTGG
PARP1	Sense: ACCACTTCTCCTGCTTCTG
	Antisense: TCTTCTCTGCCTTGCTACC
β-actin	Sense: ATCGTGCGTGACATTAAGGAGAAG
	Antisense: AGGAAGGAAGGCTGGAAGAGTG

AIF, apoptosis-inducing factor; PARP1, poly (ADP-ribose) polymerase 1.
